# Evaluation of the influence of light-curing units on the degree of conversion in depth of a bulk-fill resin

**DOI:** 10.4317/jced.57288

**Published:** 2020-12-01

**Authors:** Fernanda-Midori Tsuzuki, Lidiane-Vizioli de Castro-Hoshino, Larissa-Coelho-Pires Lopes, Francielle Sato, Mauro-Luciano Baesso, Raquel-Sano-Suga Terada

**Affiliations:** 1 Postgraduate student, Department of restorative dentistry, Dental Materials area, Campinas State University (FOP/Unicamp), Piracicaba, Brazil; 2 Postgraduate student, Department of physics, State University of Maringá (UEM), Maringá, Brazil; 3 Postgraduate student, Department of dentistry, State University of Maringá (UEM), Maringá, Brazil; 4 Associate Professor, Department of physics, State University of Maringá (UEM), Maringá, Brazil; 5 Associate Professor, Department of dentistry, State University of Maringá (UEM), Maringá, Brazil

## Abstract

**Background:**

It is known that bulk-fill have been widely studied and used by dentists in the clinic. However, the use of light-curing units that do not have the ability to adequately light-cure these materials at the appropriate depth can affect their clinical performance. The aim of this study was evaluating the influence of 5 different light curing units (LCUs) on the degree of conversion (DC) of a bulk-fill resin at depths of 0 to 4 mm and determined the effect of using 20s exposure and 40s.

**Material and Methods:**

Cylinders of composite were made in a stainless steel matrix (n=10). The specimens were exposed from the top surface using 5 LCUs: Valo® Cordless (VA); Radii Plus (RA); Emitter.D (EM), Biolux Plus (BI), Woodpecker® (WO). The emission wavelength and the power density was determined. After the photoactivation, the Raman vibrational modes were calculated taking as reference the peaks at 1,601 (aromatic bonds C=C) and 1,640 cm-1 (aliphatic bonds C=C).

**Results:**

The largest difference in DC in 20s, comparing the values obtained in the first and last layer is for BI, with a variation from 61.24% to 53.86%. Comparing the LCUs, the last layer in 40s DC values are 57.40% (BI), 58.21% (WO), 58.97% (VA), 60.90% (RA) and 62.42% (EM). The higher the dose (J/cm²) and the close the λmax is to the maximum CQ absorption length (λmax ~ 470 nm) the better the DC value.

**Conclusions:**

There was a significant difference in the DC values between the LCUs with increasing depth of the bulk-fill increments. Results indicate significant differences in DC among the different LCUs as well as enhanced DC when using 40s exposure compared to 20s. It is suggested that for DC improvement using lower power photoactivator increase the exposure time the exposure time should be 20s to 40s.

** Key words:**Polymerization, Composite Resins, Raman spectroscopy.

## Introduction

Clinical use of composite resins has become indispensable in dentistry (1). The clinical versatility of resin requires constant improvements in properties for better performance in long-term restorations, which is reflected in the continued launch of new products on the market (2,3). In 2009, bulk-fill resin was launched and the first material commercially available was Surefil® SDR resin (Dentsply Caulk, Mildford, DE, USA) (3).

Recent studies show that the bulk-fill resin has improved mechanical properties (4,5), less polymerization stress and reduced microleakage (6,7). Another advantage would be the possibility of filling the cavity in single increments of up to 4 mm, with minimal polymerization shrinkage during the photoactivation process (8), which means reducing the clinical time and reducing the risk of contamination during the restorative procedure (4). To do so, one must take into account the type of LCU used, as this may influence the depth of cure of resin composites. The monomer polymer conversion of the resins is directly related to the intensity, wavelength and irradiation time of the LCU (9).

The degree of conversion (DC) directly affects the physical and mechanical characteristics of the composite resin, influencing the durability of the restoration (10). The presence of unconverted carbon double bonds may render the material more susceptible to degradation, promoting reduced color stability and release of substances with potential for toxicity (11). Therefore, an incorrect photoactivation can cause adverse biological reactions, as well as the reduction of mechanical properties (12). In this way, adequate photoactivation is required so that light-curing resins achieve the properties desired by the manufacturer, a basic requirement for long-term predictable clinical success (13). In addition, with the introduction of different LCUs with increasing power, there is a real danger that dentists are not adequately informed about their use, increasing the number of restorative failures (14).

Among the different devices, the spectral radiant power, the active curing tip diameter and the irradiance are different, thus altering the capacity to light-cure the resins (13). Several studies (15-17) have evaluated the curing depth of bulk fill resins, but none have verified the values to their full extent when using different LCUs.

 In this context, the objective of this study was to evaluate the DC in depth of the bulk-fill resin Surefil® SDR with different LCUs. The null hypotheses tested were: (1) DC values do not change significantly with increasing depth of restoration; (2) DC in depth of a bulk-fill resin does not depend on the LCU used for photoactivation of the material and (3) DC values do not change significantly with increasing activation time.

## Material and Methods

-Experimental Design

The DC in depth was evaluated using a *bulk-fill flow* resin bulk-fill flow, Surefil® SDRTM Flow (Dentsply Caulk, Mildford, DE, USA), photoactivated in the time of 20 and 40 seconds, with 5 types of light-curing units (LCUs): Valo® Cordless/Ultradent (VA); Radii Plus/SDI (RA); Emitter.D/Schuster (EM), Biolux Plus/Bioart (BI), Woodpecker®/Guilin Woodpecker Medical Instrument (WO). The emission wavelength of each LCU was determined by a linear array spectrometer (VS140, HORIBA Jobin Yvon, Kyoto, KA, JPN). The power density of each light-curing unit was also determined (407A, Spectra Physics, Mountain View, CA, USA).

Ten specimens were prepared for each group of LCU and for each time totaling 100 specimens. The DC was evaluated by means of Raman spectroscopy after 24 hours.

-Preparation of specimens

**Figure 1:**
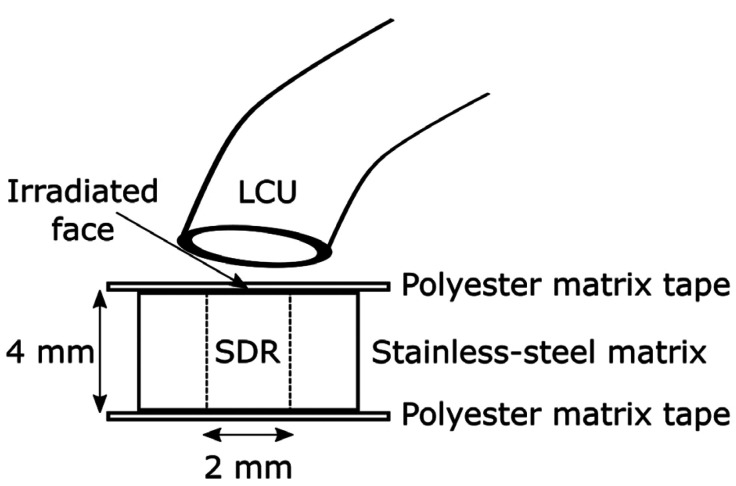
Schematic representation of specimen preparation for DC evaluation.

For the preparation of the specimens, we used a stainless-steel matrix containing a central hole measuring 2 mm in diameter by 4 mm in height. The matrix was divided in half, with lateral screws, that allow the easy removal of the specimen. The matrix was superimposed on a polyester matrix tape (TDV, Pomerode, SC, BRA) supported on a glass plate and filled with bulk-fill resin. A second polyester tape was placed with light pressure on the matrix in order to avoid excesses, obtain a regular surface and prevent contact with the surface of the light tip (Fig. 1). After the photoactivation at 20 and 40 seconds, each specimen was removed from the matrix and stored in a dry and dark environment at 36ºC for 24 hours.

-Degree of conversion assessment

All specimens were subjected to DC measurement using a confocal Raman microscope (SENTERRA, Bruker Optik GmbH, Ettlingen, BW, DEU), 24 hours after preparation. The measurements were taken with an excitation laser wavelength of 532 nm, nominal power of 20 mW, focused on the specimen by a lens of magnitude of 50x, with 3 seconds of integration time, 20 scans and spectral resolution of 9-15 cm-1 in the region between 1,778-419 cm-1. The spectra were corrected by baseline using OPUS software (7.2, Bruker Optik GmbH, Ettlingen, BW, DEU). Twenty points were read along the length of the specimen, from the face irradiated by the LCU.

-Degree of conversion calculation

In order to evaluate the DC of the bulk-fill resin, the Raman vibrational modes were calculated taking as reference the peaks at 1,601 (aromatic bonds C=C) and 1,640 cm-1 (aliphatic bonds C=C). The percentage of unconverted carbon double bonds (% C=C) was determined by the intensity rate of C=C bonds (1,601 cm-1) and C=C bonds (1,640 cm-1) before and after polymerization. The ratio between the two peaks intensity was calculated for both the polymerized and unpolymerized material. The equation used to calculate the DC was:

DC (%) = 100 x (1 - (RPolymerized /RUnpolymerized)) (1)

* R = Ratio between peaks 1,640 and 1,601 cm-1)

-Statistical analysis

DC are presented as mean ± standard deviation of the mean (SD). After tabulation of the results in a database, they were analyzed using analysis of variance (ANOVA). The Shapiro-Wilk test was used to assess the sample normality pattern. The Tukey test was used to compare the different depths and different photoactivation times. The significance level of *p* <0.05.

## Results

**Figure 2:**
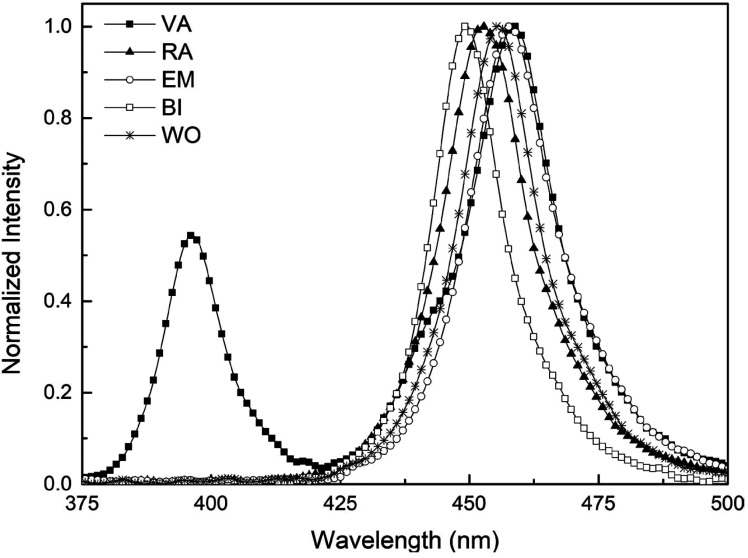
LCUs emission spectrum.

The emission spectrum of the LCUs evaluated showed that the devices work at a wavelength of 420 to 500 nm, and for VA there is a peak of light emission in the ultraviolet region (Fig. 2), wavelength region that is required to activate camphorquinone photoinitiator present in the SDR resin (13). The average power rating of each appliance is listed in Table 1.

**Table 1:**
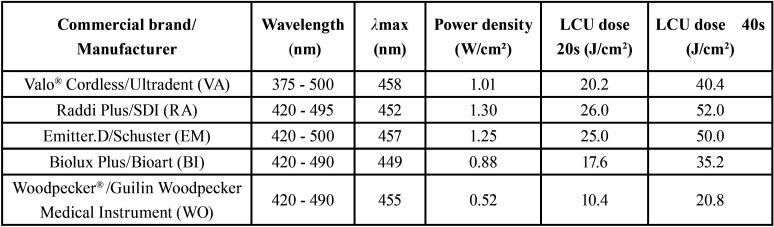
Specifications of LCUs tested in the study.

**Figure 3:**
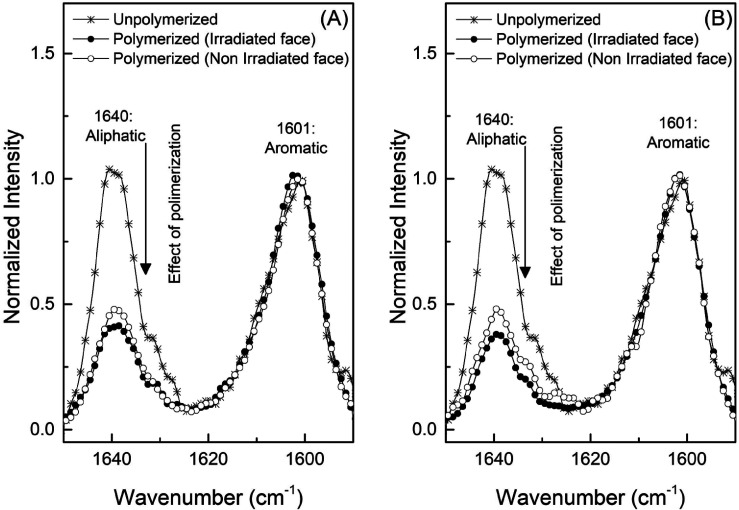
Peaks of 1,640 cm-1 (aliphatic carbon) and 1,601 cm-1 (aromatic carbon), before and after polymerization along the extension of the specimen. (A) Photoactivated for 20 seconds; (B) Photoactivated for 40 seconds.

Figure 3 illustrates the Raman spectra of the SDR resin before and after polymerization for 20 and 40 seconds. Note the polymerization effect by decreasing peak intensity at 1,640 cm-1; this change is associated with the formation of the polymeric structure.

Table 2 presents the significant differences in DC between photoactivators and depths. Comparing the devices, at the time of photoactivation of 20s, the first depth layer of the resin presented homogeneous DC values for all LCUs. However, in 1-2 mm BI shows the lowest DC values. From 2 mm, BI, WO and RA presented the lowest values. In 40s time, BI showed statistical differences between all photoactivators at all resin depths. In the last layer, DC values in ascending order are 57.40% (BI), 58.21% (WO), 58.97% (VA), 60.90% (RA) and 62.42% (EM).

**Table 2:**
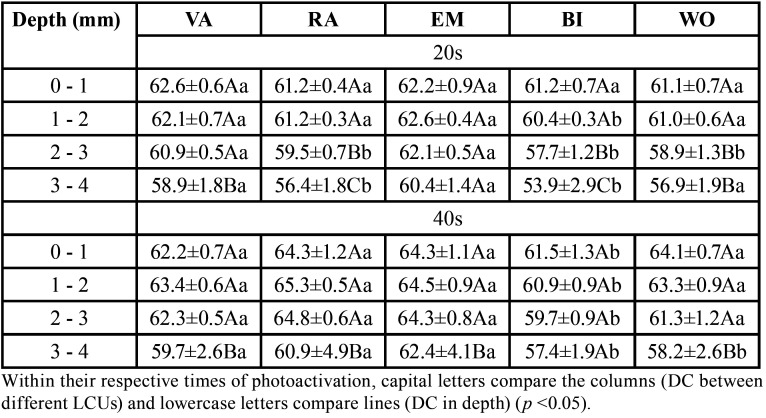
Mean ± SD for ranges within 1 mm thick increments of the degree of conversion (%) of bulk-fill SDR resin at different depths and different photoactivation times (values measured every 0.2 mm in figure 4 were summed and the average was obtained for each mm depth).

In general, within the same photoactivator, the layers presented statistical differences from 3mm. The largest difference in DC in 20s, comparing the values obtained in the first and last layer is for BI, with a variation from 61.24% to 53.86%. In the 40s, the WO photoactivator is responsible for the greatest variation between the first and last layers, with 64.11% and 58.21%, respectively.

**Figure 4:**
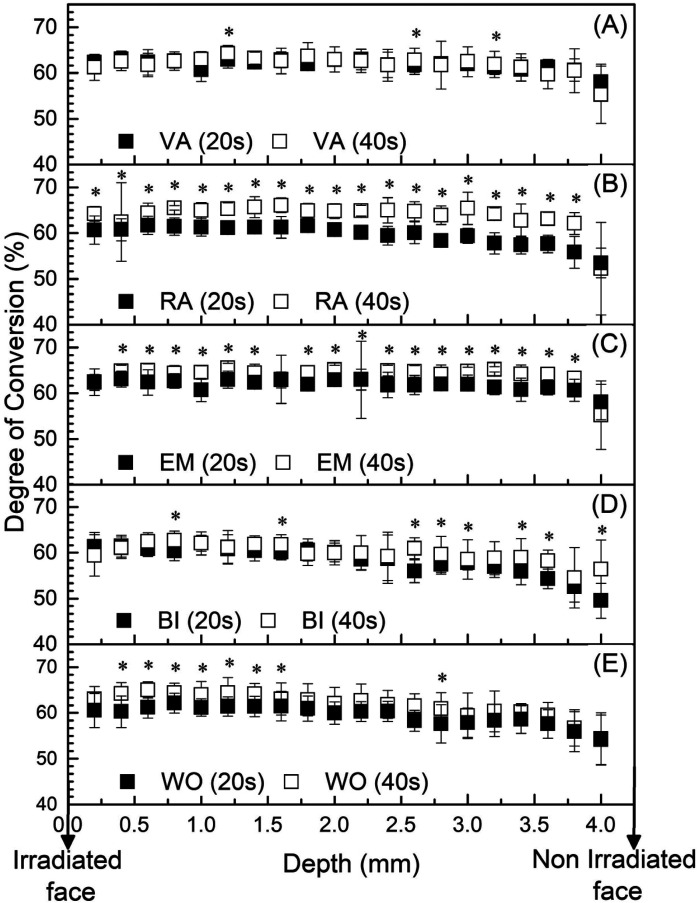
Mean ± SD of DC (n=10) measured every 0.2 mm for each LCUs: (A) VA; (B) RA; (C) EM; (D) BI e (E) WO (letter a shows statistical difference between 20 seconds and 40 seconds *p* < 0,05).

Figure 4 shows the significant differences in CG measured every 0.2 mm at different times. The increase from 20s to 40s in the photoactivation time of the resin results in the increase of DC, with the exception of the VA photoactivator that maintained the DC similar when comparing the times. The RA obtained the greatest statistical differences comparing the two times of photoactivation.

## Discussion

The null hypotheses were rejected because the DC values varied significantly according to the increase in the depth of the material, the LCU and the time of photoactivation. As the light absorption of photoinitiators is essential to improve the efficiency of the photochemical reaction, it is important to select resinous compounds with absorption spectra that overlap the emission spectra of the irradiation sources. The literature describes that in order to improve the photoactivation depth of bulk-fill resins, several characteristics have been introduced into their composition (6,16,19), which provides a more uniform conversion of monomers in depth (20-22).

According to the manufacturer, resin has a photosensitive molecule called camphorquinone (CQ) and a new UDMA-based monomer that reduces resin shrinkage stress (10,23). The results showed that the LCUs exhibited 420-490 nm emission peak, coinciding with the maximum absorption peak of the CQ (24).

The emission peak in the ultraviolet region found in Valo (Fig. 2) is because it is a polywave device. For bulk-fill resins containing only CQ as a photoinitiator, as in the resin case, the monowave and multywave LEDs have shown the same efficiency (1,24,25). For composites containing CQ associated with alternative photoinitiators, the polywave LED has higher DC because these alternative photoinitiators require shorter wavelengths (26). Corroborating the literature, it was observed that the ultraviolet peak did not show strong influence on the polymerization of the resin used. However, it is important for the clinician to have a device with two or more emission peaks for use with other resinous materials that have modern photoinitiators (13).

To analyze the DC, different methods have been proposed such as microhardness (8,20), ISO 4049 scraping (27), Fourier transform infrared spectroscopy (FTIR) (5), and vibrational spectroscopy, such as Raman spectroscopy and FTIR, are considered more accurate because they directly quantify the number of unreacted C=C bonds (28,30). The main advantage of Raman spectroscopy is to work with the samples in a non-destructive way, which allows multiple measurements in the same sample (10). Differently from previous work evaluating DC only on upper and lower surfaces, this study mapped the DC along the resin by performing a 20-point reading on the entire specimen.

Data show that the LCUs presented differences between them because, although the first layer of resin presented homogeneous DC values for all the LCUs, in the last layer of depth the values between the devices varied from 53.87% to 60% in the time of 20s and from 57.40% to 62% within 40s. To date, the minimum DC for clinically satisfactory restoration has not been established accurately. In the time suggested by the manufacturer of 20s, the EM and VA devices presented better results values close to 60%, unlike the WO and RA that were close to 56% and the BI device that presented even lower values.

The results suggest that the dose (J/cm²) and the maximum emission wavelength (λmax) of the LCUs influences the DC (Table 1). The higher the dose and the close the λmax is to the maximum CQ absorption length (λmax ~ 470 nm) the better the DC value. The LCUs EM and VA, which presented better results in the time suggested by the manufacturer, have λmax = 457 and 458 nm, with exposure of 25.0 and 20.2 J/cm², respectively. The BI that presented the lowest values has λmax = 449 nm with exposure of 17.6 J / cm2.

However, the study showed that the increase in photoactivation time may increase DC values. This is because during the activation of the resin, the photons activate the initiator and generate free radicals that start the polymerization, presenting a synergistic effect on the rate of polymerization (26). The literature shows that the optical properties of resin composites and their light activated polymerization reactions are interdependent: greater radiant exposure produces a higher degree of conversion (29). In this way, it is possible to extend the photoactivation time to achieve a higher degree of conversion.

Nevertheless, it is worth emphasizing the limitations of this study. Because it is an *in vitro* study, the DC may have presented better results because if the restorations were performed clinically, the distance from the cusps, incorrect tip orientation and limitation of mouth opening may interfere with the amount of light that will arrive in the deeper layers (30). In this way, it is necessary for the professional to be sure that the device is in adequate conditions for an effective polymerization (14), and this can be done by routinely monitoring the light output of the LCU through a radiometer (30).

Today, LED-based sources predominate in dental offices (29). Therefore, these results should have an impact on the clinical daily life, since the professional must pay attention to the depth of preparation in which material will be inserted, the photoactivation time of the resin and the type of LCU used, in order to increase the clinical life of restorations and decrease sensitivity, microleakage and caries recurrence.

## Conclusions

In summary, there was a significant difference in the DC values between the LCUs and with increasing depth of the resin increments. It was also observed that the increase in the time of LCUs results in increased DC of the resin. It is suggested that for DC improvement using lower dose photoactivators increase the exposure time from 20 to 40s.
